# Donation after circulatory death; cholangiopathy in the machine age

**DOI:** 10.1097/MOT.0000000000001222

**Published:** 2025-05-21

**Authors:** Ian S. Currie, Fiona M. Hunt

**Affiliations:** a Edinburgh Transplant Centre; b Institute for Regeneration and Repair, University of Edinburgh; c NHS Blood and Transplant, UK

**Keywords:** cholangiopathy, donation after circulatory death, liver, nonanastomotic strictures, outcome

## Abstract

**Purpose of review:**

Published work evaluating machine perfusion of DCD (donation after circulatory death) liver grafts in situ and ex situ is rapidly evolving, with several landmark studies published in the last 6 months. The central question in DCD liver transplant remains; which strategies most effectively reduce cholangiopathy? This condition, which results in repeated hospital admissions, interventions, re-transplantation and death, is a major deterrent to DCD utilization. This review considers current evidence in the mitigation of transplant cholangiopathy by machine perfusion in DCD liver grafts.

**Recent findings:**

Studies which directly address DCD cholangiopathy as a primary outcome are few in number, despite their critical importance. In systematic reviews, Normothermic Regional Perfusion and Hypothermic Machine Perfusion consistently and significantly reduce transplant cholangiopathy rates. By contrast, the efficacy of Normothermic Machine Perfusion performed at donor or recipient centres is less well described and cautious interpretation is required. The most recent development, namely hypothermic followed by normothermic perfusion, has only now appeared in the literature but appears to offer advantages compared to either technology alone.

**Summary:**

To reduce DCD cholangiopathy, current data best support the use of donor centre NRP or recipient centre HMP. However, utilization is also improved when warm perfusion is involved.

## INTRODUCTION

Transplant cholangiopathy is a multifocal stricturing disorder of the graft biliary tree with a patent artery [1], most commonly seen in donation after circulatory death (DCD) grafts, and strongly associated with recurrent sepsis, multiple interventions, graft failure and death. Despite risk factors such as cold ischemic time (CIT) > 8 h and donor age >40 being long recognized as significantly associated with cholangiopathy [2], hazard ratios in a 2011 meta-analysis showed no evidence of improvement over a decade [3]. Persisting poor outcomes [4–6] confirmed the promise of cholangiopathy-free DCD liver transplantation would only be realized by addressing the challenges of DCD liver preservation.

This review describes outcomes of machine perfusion and the amelioration of transplant cholangiopathy in selected literature. Data show clearer associations for DCD biliary injury with subsequent nonanastomotic strictures (NAS), where this work is focussed. The terms cholangiopathy and NAS are used interchangeably. 

**Box 1:**
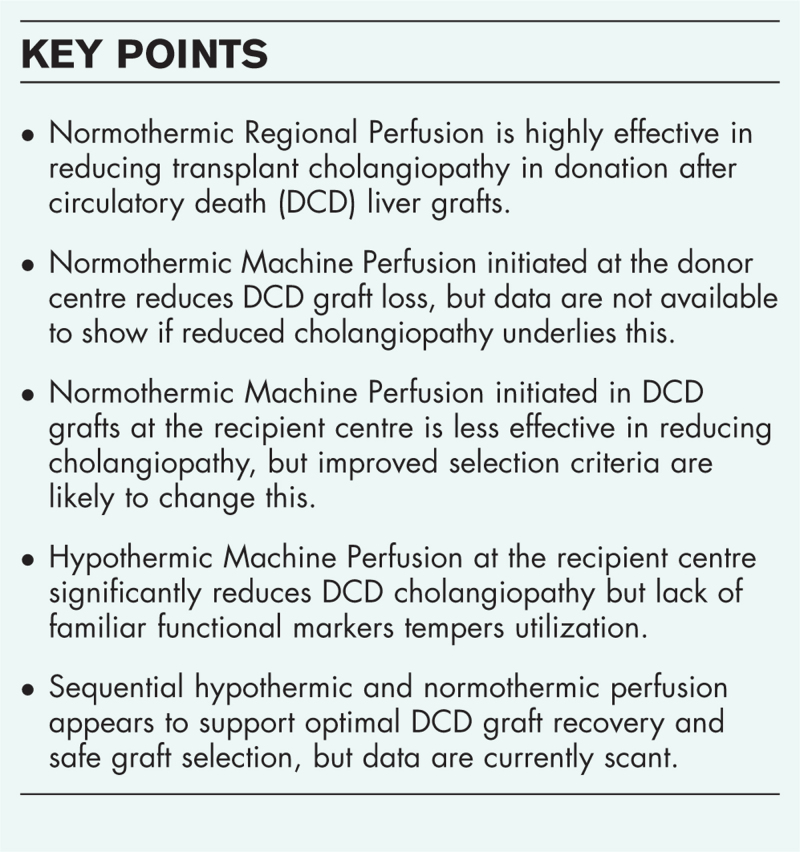
no caption available

**FIGURE 1:**
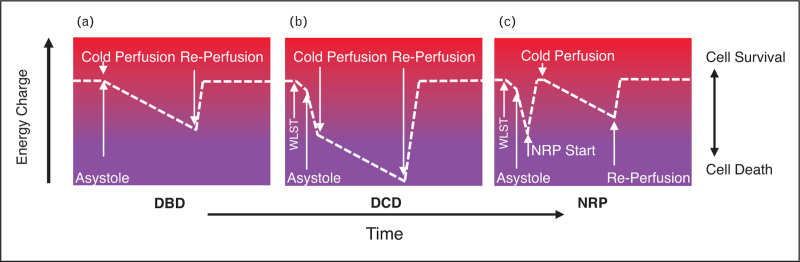
Energy charge during liver retrieval and preservation. A. In DBD organ donation, cold preservation supervenes immediately after warm perfusion. There is no significant ischemic phase. Energy charge depletes slowly during cold storage prior to re-perfusion in the recipient. If cold ischemic time is too long, severe injury may occur, reducing viability in the recipient. B. In DCD livers, there is rapid energy depletion during the peri-mortem and asystolic phases. Cold preservation arrests this rapid phase, but the liver enters cold storage more energy-deplete than in DBD. This means that cold ischemic tolerance is less than for DBD. C. In NRP livers, the rapid energy depletion of the perimortem DCD phase is restored to normal, so that the liver enters cold storage much like a DBD liver. Figure 1 has appeared previously in presentations at the annual meeting of the International Liver Transplant Society (May 2024,Houston) and at the British Transplant Society's Annual Monothematic meeting (November 2023, London).

**FIGURE 2:**
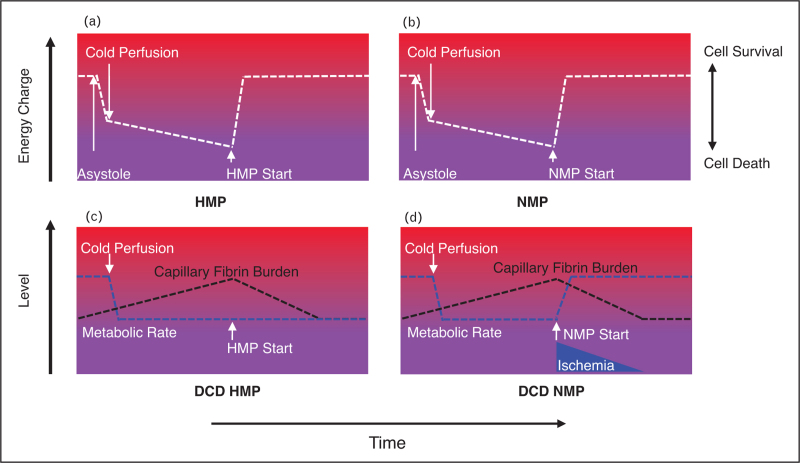
Fibrin deposition and biliary injury in HMP and NMP. A/B. After DCD organ donation and cold storage, evidence suggests that use of HMP or NMP restores energy charge in both. On this basis, there is no difference between HMP and NMP, which is not born out by the higher cholangiopathy rates in NMP livers. Progressive fibrin deposition in the peribiliary plexus is likely to be the additional factor which explains this. C. Circulatory standstill in peri-mortem DCD donor livers is associated with fibrin deposition in graft capillaries [27,28]. Two hours of HMP most likely flushes out fibrin deposits before tissues become metabolically active again at re-perfusion. This means the peribiliary plexus is once again patent at the time of warm re-perfusion, reducing secondary ischaemic injury. D. If NMP (or transplant re-perfusion) rather than HMP is initiated after cold storage, there is insufficient time to flush out the peribiliary plexus before the metabolic demand of the normothermic biliary tissue rises sharply. In the face of inadequate peribiliary perfusion secondary to persisting fibrin, there is an added biliary ischaemic injury, leading to cholangiopathy. It is likely that NRP, which begins sooner after asystole than all other perfusion techniques, flushes out early fibrin deposits or may prevent these deposits forming, as fibrin levels in NRP livers are lower than DBD and DCD [27,28]. It may be that the dramatic effects of NRP are a result of both favourable metabolic enhancement and reduced fibrin burden.

The following sections describe machine perfusion strategies in use, divided by location (donor or recipient centre), their effects on cholangiopathy, risk stratification and consequences for utilization. A tabulation of results is given in Table 1 and illustrated in Figures 1 and 2.

**Table 1:** Summary table of cholangiopathy in machine perfusion studies

Reference	Perfusion	Cholangiopathy	^∗^Graft loss (perfusion versus SCS)
9; UK; R	NRP	0/42 vs. 47/171 (*P* < 0.0001)	97.7% vs. 89.8% (*P* = 0.1019)
14;US;R	TANRP	0/104 vs. 12/133 (*P* = 0.002)	*P* = 0.37
13; EU; R	NRP	6/545 vs. 24/258 (*P* < 0.001)	77/545 vs. 88/258 (*P* < 0.001)
15;SR	NRP	13/702 vs. 68/505 (*P* = 0.0002)	17/702 vs. 22/505 (*P* = 0.04)
16;UK;PR	DCNMP	3/27 vs. 5/19 (*P* = 0.18)	NR
19;EU;R	DCNMP	NR	HR 0.13 (*P* < 0.001)
20;EU;R	RCHMP	0/25 vs. 11/50 (*P* = 0.013)	90% vs. 60% survival (*P* = 0.002)
21;EU;PR	RCHMP	RR 0.36 (*P* = 0.03)	PNF 0/78 vs. 1/78
23;SR	RCHMP	OR 0.25 (*P* < 0.01)	OR Survival 2.17 (*P* = 0.02).
24;UK;R	RCNMP	RR 0.82 (*P* = 0.19)	HR 0.9 (*P* = 0.69)
26;US;R	RCNMP	2/37 vs. 1/74 (*P* = 0.051)	1/37 vs. 1/74 (*P* = 0.643)
23;SR	RCNMP	OR 0.64 (*P* = 0.21).	NR
			
23;SR	1xDCNMP+1xRCNMP	OR 0.64 (*P* = 0.21)	NR
31;US;R	NRP vs. NRP+DCNMP vs. SCS	0/62 vs. 0/21 vs. 50/297 NRP vs. SCS *P* < 0.001 NRP+NMP vs. SCS *P* = 0.04	NR
32;EU;P	RCHMP+NMP	1/34	NR

DC, donor centre; HR, hazard ratio; NR, not reported for DCD subgroup; OR, odds ratio; P, prospective; PR, prospective randomized; R, retrospective; RC, recipient centre; RR, relative risk; SR, systematic review.

∗ Graft loss; some results were provided as Graft Survival as indicated. Machine perfusion is the reference for both cholangiopathy and graft loss.

## PERFUSION TECHNIQUES AT THE DONOR CENTRE

### Donor centre; normothermic regional perfusion (NRP)

The earliest reperfusion of ischaemic donor organs is provided by NRP, which uses an extra-corporeal circuit to provide warm oxygenated blood to a restricted part of the donor body [7–9] followed by static cold storage (SCS) for transport. Abdominal NRP (ANRP) refers to perfusion of organs below a clamp placed on the descending thoracic aorta, whereas thoraco-abdominal NRP (TANRP) perfuses all organs excluding the brain and head [10,11]. ANRP is referred to here.

Fully accredited surgeons are generally required to initiate an NRP program [12], given the rapidity and gravity of decision making required. However, competent senior Fellows can be trained to this role. Save for venous cannulation, and a vent in the aortic arch to confirm no cerebral perfusion, surgery is no different from standard DCD. Perfusion-wise, advanced practitioners can be trained as perfusion specialists from backgrounds other than cardiopulmonary bypass [12]. Although organ resuscitation occurs rapidly, a two hour period of NRP is generally used to allow blood tests for organ quality assessment [7–9,13].

In a retrospective UK study, 43 NRP livers were compared with 187 SCS DCD grafts [9]. Graft cholangiopathy occurred in 0/42 NRP livers vs. 47/171 SCS DCD livers (*P* < 0.0001). PNF (primary non-function) occurred in 0/42 NRP grafts and 13/187 SCS grafts (*P* = 0.13). Graft survival censored for death was not significantly different at 90 days; 97.7 [95% confidence interval (CI): 84.6–99.7] in the NRP group against 89.8 (95% CI: 84.5–93.4) in the SCS group (*P* = 0.10), although 90 days is too early to assess graft loss from cholangiopathy.

In a 2024 U.S. study, 106 NRP were compared with 136 SCS DCD. Although most NRP livers were recovered using TANRP, and not strictly comparable with other studies described here, it is noteworthy that 0/104 NRP vs. 12/133 SCS DCD transplants developed NAS (*P* = 0.002) [14^▪▪^].

In the largest retrospective series published so far, graft cholangiopathy was present in 6/545 NRP livers (1%) against 24/258 (9%) of SCS DCD transplants [hazard ratio (HR) = 0.112 (95% CI: 0.042–0.299); *P* < 0.001] [13]. Graft loss, not censored for death, was 77/545 (14%) vs. 88/258 [34%; HR = 0.371 (0.267–0.516); *P* < 0.001]. Death was 65/545 (12%) vs. 66/258 [26%; HR = 0.540 (0.373–0.781) *P* = 0.001], favouring NRP. Notably, only CIT [HR = 1.004 (1.001–1.007); *P* = 0.021] and re-transplant [HR = 9.552 (3.519–25.930); *P* < 0.001] were significant causes of NRP graft loss in multivariate analyses.

The most recent systematic review and meta-analysis included 4 studies of NRP vs. SCS DCD. Cholangiopathy was detected in 13/702 NRP transplants against 68/505 SCS DCD liver transplants [risk ratio (RR) 0.23 (95% CI: 0.11–0.49) *P* = 0.0002]. PNF occurred in 17/702 vs. 22/505 [RR = 0.51 (95% CI: 0.27–0.97) *P* = 0.04]. The risk of death (pooled hazard ratios) was 0.5 (95% CI: 0.36–0.69, *P* < 0.0001) [15^▪▪^].

All told, data confirm that ANRP dramatically reduces DCD cholangiopathy and PNF whilst increasing graft and patient survival.

### Donor centre; normothermic machine perfusion

Donor centre NMP is ‘simpler’ than NRP. However, expert graft preparation prior to warm perfusion is required, as well as a technologist to operate the device. The liver is cannulated via artery, portal vein and bile duct, plus or minus cava. Devices aim to support graft resuscitation over at least 2–4 h as required to allow assessment of viability parameters.

In a randomised prospective study of 222 patients transplanted with DBD (*n* = 167) and DCD (*n* = 55) grafts treated with SCS or NMP at the donor hospital, MRCP was performed per protocol on 46 of the 55 DCD (34 NMP; 21 SCS) recipients [16]. Cholangiopathy occurred in 3/27 DCD NMP grafts and 5/19 DCD SCS livers (*P* = 0.18). Re-transplant for cholangiopathy was required in one patient for each arm (121 NMP; 101 SCS). Graft losses were not reported at the level of graft type.

In the randomized PROTECT study, cholangiopathy overall was present at one year in 4 of 151 (2.6%) in the NMP group and 14 of 142 (9.9%) in the SCS group (*P* = 0.02) [17]. However, the study group contained only 39 DCD grafts from 293 transplants. Parallel work using a different device in a prospective randomized U.S. study of 136 NMP and 131 SCS livers (DBD and DCD) showed the reported rate of cholangiopathy was very low indeed (2 cases in NMP; 1 in SCS) [18]. However, only 22/136 in the NMP and 16/115 in the SCS groups were DCD livers, and cholangiopathy was not a study outcome. Graft survival was 97% for NMP and 97.7% for SCS. Sadly, few if any conclusions can be drawn as regards DCD NAS in these otherwise critically important studies.

A large, single-centre retrospective study included 1086 DBD and DCD grafts receiving NMP or SCS (DCD SCS = 264; DCD NMP = 279; DBD SCS = 480; DBD NMP = 63) [19]. A primary outcome measure was EAD, and secondary outcomes included graft survival at one year. EAD was lower for DCD-NMP grafts (17.5%) than in DCD-SCS (50.0%; *P* = 0.002). There was an 87% decrease in DCD death-censored graft failure in those grafts treated with NMP (HR, 0.13; 95% CI: 0.05–0.33; *P* < 0.001). Unfortunately, cholangiopathy was not recorded.

At best, these studies show that the quality of evidence around abrogation of DCD cholangiopathy by Donor Centre NMP is weak. The absence of subgroup analysis and the conflation of DBD and DCD in these studies, grafts with very different risk profiles, is challenging.

## PERFUSION TECHNIQUES AT THE RECIPIENT CENTRE

### Recipient centre; hypothermic machine perfusion

Hypothermic machine perfusion (HMP) is considered by some to be ‘safer’ than warm perfusion as perfusion failure does not necessarily lead to organ loss. Expertise required is modest, with cannulation of portal vein alone reported in most studies. Perfusion times reported are approximately 2 h [20,21,22^▪▪^].

The first human study was a retrospective comparison of HOPE (Hypothermic Oxygenated Perfusion; *n* = 25) with SCS (*n* = 50). Despite greater functional warm ischaemic time (FWIT) in HOPE livers [median (IQR); HOPE = 31 mins (26–36); SCS = 26 mins (20–29); *P* < 0.0001], cholangiopathy occurred in 0/25 HOPE grafts and in 11/50 SCS livers (*P* = 0.013) [20]. PNF occurred in 0/25 HMP and 3/50 SCS livers (*P* = not significant). One year graft survival was superior in HOPE (90%) compared to SCS (69%; *P* = 0.002).

Dual HOPE (DHOPE; perfusion via hepatic artery and portal vein) was used in the only HMP prospective randomized study comparing DHOPE (*n* = 78) and SCS (*n* = 78) groups. Despite the longer preservation time in HMP grafts [(median (IQR)] of 524 (466–554) minutes for DHOPE as against 409 (356–477) min for SCS, DHOPE reduced symptomatic nonanastomotic strictures (trial MRCP at 6 months if not before) by two-thirds (RR, 0.36; 95% CI: 0.14–0.94; *P* = 0.03). PNF occurred in 0/78 and 1/78 grafts in the HMP and SCS groups [21]. Despite this promising result, supplementary trial data revealed that the proportions of mild, moderate and severe cholangiopathy as determined by trial MRCP were not different between the HMP (mild 49%; moderate 35%; severe 16%); and SCS groups (mild 58%; moderate 32%; severe 10%; *P* = 0.64).

In a systematic review, NAS risk was significantly decreased by HMP [odds ratio (OR) 0.25 95% CI 0.12–0.51, *P* < 0.01] compared to SCS [23]. One-year graft survival was significantly improved in HMP (OR 2.17, 95% CI: 1.14–4.11, *P* = 0.02), however, 1-year patient survival was not different 92% (127/138) vs. 93% (137/148; OR 1.38, 95% CI: 0.21–9.09, *P* = 0.74).

It is reasonable to conclude that HMP significantly reduces cholangiopathy by up to three quarters, and significantly reduces graft loss, compared to SCS.

### Recipient centre; normothermic machine perfusion

A prospective multicentre study of RCNMP in DBD (*n* = 23) and DCD (*n* = 8) grafts compared these with a dataset gathered during parallel UK donor centre normothermic machine perfusion (DCNMP) studies (*n* = 104), thereby determining if delayed onset of NMP until arrival in the Recipient Centre produced different outcomes [24]. Recipient and Donor Demographics were not significantly different in the groups. Perfusion parameters were generally not different, although arterial flow rates were 50% higher in the RCNMP study group (*P* < 0.001). Outcomes, not divided by graft type, were similar although it was notable that 1 year graft survival was 84% in the study group (RCNMP) vs. 94% in the DCNMP group (*P* = 0.08).

A single-centre retrospective study examining outcomes from DCD liver transplants after SCS (*n* = 97), RCNMP (*n* = 67) and NRP (*n* = 69) [25]. Multivariate, risk-adjusted logistic regression showed that risk of cholangiopathy was 0.82 (0.34–1.98) in NMP compared to SCS (*P* = 0.19). 4/67 and 6/97 grafts were lost to cholangiopathy in NMP and SCS groups, giving a hazard ratio of 0.9 (95% CI 0.4–1.7; *P* = 0.69) for cholangiopathy-specific graft loss in NMP vs. SCS.

A ‘real world’ U.S. retrospective cohort study containing (37 NMP + 74 SCS) DCD and (118 NMP + 236 SCS) DBD liver transplants aimed to assess healthcare costs and complications as primary outcomes between SCS and NMP [26]. Cholangiopathy was diagnosed in 2/37 DCD NMP livers and 1/74 DCD SCS livers (*P* = 0.051), with graft loss of 1/37 and 1/74 (*P* = 0.643) at 90 days. The study was not designed with NAS as a primary outcome, and 90 days is too early to conclude NAS rate in DCD grafts which generally arise over 12 months. That aside, this study provided no evidence for benefit of RCNMP in DCD cholangiopathy. It did confirm that the added costs of machine recovery were fully offset by the lower burden of healthcare costs in RCNMP liver transplant.

It appears there is no evidence of benefit regarding RCNMP minimizing DCD cholangiopathy. However, Watson *et al.* have highlighted the presence and effect of fibrin deposition in liver grafts [27,28], likely as a consequence of activated coagulation in organ donors [29,30]. Fibrinolysis during NMP may have a major role to play in resolving the therapeutic contribution of RCNMP to minimization of DCD cholangiopathy.

## COMBINATIONS OF PERFUSION TECHNIQUES

### Sequential perfusion technologies

Retrospective comparisons were made between 62 NRP grafts, 21 NRP+DCNMP grafts and 297 SCS livers in a study where TANRP exceeded ANRP as the NRP technique. Donor and recipient demographics were not meaningfully different, although total perfusion time was significantly longer in NMP livers. Timings suggested that most NMP began at the donor centre. Cholangiopathy was diagnosed in 0/62 and 0/21 patients in the NRP and NRP+NMP groups respectively, but in 50/297 of the SCS group (16.8%; vs. NRP; *P* < 0.001; vs. NRP+NMP; *P* = 0.04) [31^▪▪^].

HMP plus NMP was utilized in 54 initially discarded livers in a prospective study. DCD grafts (plus 1 DBD) were recovered in SCS and placed on RCHMP for 60 min, followed by ‘controlled re-warming’ to NMP for at least 2.5 h to undergo viability assessment according to the group's previously published criteria [32–34]. Donors were no different between groups, except for a higher DRI in the nontransplanted group. 34/54 met both hepatocellular and cholangiocyte criteria on NMP and all were transplanted, with 1/34 developing NAS (radiological signs and clinical signs or symptoms) by 12 months posttransplant.

## STRATIFICATION OF RISK

There are no published studies which address cholangiopathy risk stratification in NRP-treated grafts prior to transplant. Possibly, this relates to very low cholangiopathy rates for NRP livers in recent studies (1% [13] or 0% [31^▪▪^]). It seems unlikely that estimation of cholangiopathy risk during NRP will be addressed prospectively. Risk assessment for DCD HMP livers developing NAS has been postulated in a recent multicountry study [35^▪▪^]. The data are comprehensive, yet offer a receiver operator characteristic (ROC) curve area under the curve (AUC) of 0.756 (95% CI: 0.6881–0.8030; *P* < 0.0001) based on perfusate FMN levels to predict cholangiopathy. Although this is a step forward, it may not be discriminatory enough for lower volume centres or those new to HMP.

RCNMP studies have used bile chemistry to select grafts at lower risk of NAS [32,36]. This is area is developing rapidly, and refined criteria are likely to emerge. In 2022, the Cambridge group described 11/84 DCD grafts developing NAS after satisfactory RCNMP performance. They have since reflected that graft selection on NMP does not yield a binary output, expanding this point in a recent publication [37^▪▪^]. A parallel study from the Groningen group in initially discarded DCD livers assessed on RCNMP after HMP resulted in 34/54 livers being transplanted, of which only 1 developed NAS [32]. It should be borne in mind that the pretest probability of NAS is reduced by HMP. Therefore, selection on NMP after HMP may appear prescient. This is a highly significant study nonetheless, as it demonstrates important increases in the safety margin and utilization of declined DCD grafts. A prospective study to assess DCHMP+NMP vs. HMP or NMP would be very well received.

## UTILISATION

Warm perfusion technologies provide familiar data relating to function, such as lactate/glucose profiles, and bile chemistry. Utilization of DCD grafts is increased with NMP (NMP utilization 28/55 (51%) vs. DCD SCS utilization 13/51 (25%); *P* = 0.007 [17]; NMP 70% vs. SCS 0% [38]) as it is with NRP (increased odds of a liver offer resulting in a transplant vs. SCS; 2.904 (95% CI: 2.083–4.048; *P* < 0.0001) [39].

HMP does not generate functional data in the same way as NRP/NMP. Levels of the injury marker Flavin Mononucleotide (FMN) in perfused liver effluent have been used to support selection of grafts suitable for transplantation. Paradoxically, this reduced overall utilization in a large retrospective study [40], however PNF and NAS rates were acceptable, and higher acceptance rates followed by selective utilization may achieve good program outcomes.

It is fair to observe that large centres familiar with HMP are able to utilize higher proportions of HMP livers, whereas newer centres are naturally cautious in the absence of familiar functional data. Developments will occur when truly functional data are available in HMP, or HMP data are tightly correlated with subsequent functional data in NMP or posttransplant.

## CONCLUSION

This review has considered the most up-to-date sources to determine how machine perfusion can minimize DCD cholangiopathy, accepting that the literature is very much in evolution. Data show that NRP and HMP reduce cholangiopathy significantly. Counterintuitively, the evidence for NMP is much less clear, although this is likely to change soon. Whilst warm techniques best support utilization, a prospective study of DCD NMP focussed on cholangiopathy would be a welcome addition to the research base.

The literature shows many opportunities. There is a strong confirmation bias in the numerous retrospective studies, as surgeons only transplant those livers they believe show satisfactory perfusion parameters. Large, prospective NMP studies have not prioritized cholangiopathy as a primary outcome and have conflated DBD and DCD grafts. Many studies use EAD as an outcome, which depends partly on recipient transaminase levels which are washed out of the graft during preoperative machine perfusion. EAD is therefore not a reliable index in perfusion studies, which should focus on graft function rather than graft injury.

In conclusion, there is now good evidence that cholangiopathy can be substantially reduced by machine perfusion, and that DCD liver transplant is growing into the major opportunity so much hoped for decades ago. To maximize this opportunity, there are hard miles ahead whilst selection criteria on machine perfusion are refined and tightly correlated with functional and biliary markers in the postoperative graft.

## Acknowledgements

*None.*

### Financial support and sponsorship

*None.*

### Conflicts of interest

*There are no conflicts of interest.*

## REFERENCES AND RECOMMENDED READING

Papers of particular interest, published within the annual period of review, have been highlighted as:

▪ of special interest
▪▪ of outstanding interest
